# Beyond Traditional Repair: Comparing eTEP and Open Sublay for Ventral Hernia Repair

**DOI:** 10.3390/jcm14082586

**Published:** 2025-04-09

**Authors:** Phillip Looft, Fadl Alfarawan, Maximilian Bockhorn, Nader El-Sourani

**Affiliations:** 1 Department for Pediatric Surgery, University Hospital Oldenburg, Klinikum Oldenburg AöR, Rahel-Straus-Straße 10, 26133 Oldenburg, Germany; 2 Department for General and Visceral Surgery, University Hospital Oldenburg, Klinikum Oldenburg AöR, Rahel-Strauß-Straße 10, 26133 Oldenburg, Germanymaximilian.bockhorn@uni-oldenburg.de (M.B.); 3 Department for General-, Visceral-and Transplant Surgery, Universitätsklinikum Münster, Waldeyerstraße 1, 48149 Münster, Germany

**Keywords:** eTEP, open sublay, ventral hernia, outcomes, ventral hernia repair

## Abstract

**Background:** Ventral hernias are common abdominal wall defects requiring surgical repair to prevent complications. This study compared two techniques: minimally invasive enhanced-view totally extraperitoneal (eTEP) approach and the open sublay (OS) method, historically regarded as the gold standard. **Methods:** A retrospective single-center study was conducted between July 2019 and March 2023 at the Department for General and Visceral Surgery, Klinikum Oldenburg. All patients who underwent either eTEP or OS for ventral hernia repair were included. Patient demographics and perioperative data were collected and compared. **Results:** A total of 139 patients were analyzed, with 92 undergoing eTEP repair and 47 undergoing OS. Both groups were comparable in demographic and clinical characteristics. Significant differences were found in defect size (median 6 cm^2^ for eTEP vs. 16 cm^2^ for OS, *p* < 0.028) and mesh size (median 450 cm^2^ for eTEP vs. 150 cm^2^ for OS *p* < 0.001). Operative time (*p* = 0.119) and postoperative pain levels over 3 days showed no significant differences (VAS Day1 *p* = 0.884; VAS Day3 *p* = 0.636). Intraoperative complications were 2.17% for eTEP and 6.38% for OS (*p* = 0.207). Postoperative complications (6.52% vs. 21.28%, *p* = 0.009) and hospital stay (median 3 days vs. 5 days, *p* < 0.001) were significantly lower in the eTEP group. **Conclusions:** eTEP is a safe, effective procedure and appears to offer more advantages than OS for ventral hernia repair. It is associated with a significantly lower complication rate, as well as shorter hospital stay.

## 1. Introduction

Ventral hernias, encompassing a range of anterior abdominal wall defects, present a significant clinical challenge, affecting a substantial portion of the global population; therefore, they are one of the most common procedures in general surgery [1]. Ventral hernias frequently occur due to increased intra-abdominal pressure, which can be caused by factors such as obesity, chronic cough, postoperative changes, or even genetic predispositions. The risk factors include high body mass index (BMI), advanced age, gender, and concomitant chronic diseases, such as diabetes and cardiovascular conditions. Traditionally, ventral hernias are diagnosed using a combination of clinical examinations and imaging techniques like ultrasound or CT scans. Their management is crucial for relieving patient discomfort and preventing complications, such as incarceration and strangulation [1,2]. Over the years, various surgical techniques have been developed and refined to repair ventral hernias, each aiming to reduce recurrence rates, minimize postoperative pain, and improve patient outcomes [3].

The open sublay (OS) method, a well-established variant of retromuscular hernia repair, has traditionally been regarded as the gold standard for ventral hernia repair [4]. This approach involves placing a mesh between the posterior rectus sheath and the rectus muscle, ensuring robust support in a well-vascularized area, which promotes mesh integration and healing. The OS method’s reputation as a durable and reliable repair derives from its effectiveness in reinforcing the abdominal wall and minimizing recurrence, making it particularly useful for complex or larger hernias. Previous work by Bittner et al. and Deerenberg et al. supports the durability of the OS approach, especially for cases requiring substantial mesh support [4,5]. Furthermore, Köckerling et al. and the European Hernia Society (EHS) guidelines emphasize the OS approach’s efficacy and long-standing use in hernias with challenging anatomical features [6,7].

More recently, the eTEP approach has gained popularity, especially in scenarios where a minimally invasive method is preferred [8]. Dr. Igor Belyansky and colleagues are often credited with pioneering the eTEP technique in the early 2010s as a minimally invasive approach to ventral hernia repair that avoids entering the peritoneal cavity, thus reducing the risk of adhesions and postoperative complications and improving recovery time [8]. This innovation has positioned the eTEP as an attractive alternative when minimizing invasiveness is a priority, in tandem with advancements in surgical technology and laparoscopic expertise. The technique involves a precise extraperitoneal dissection that allows for optimal visualization of the anatomical structures while minimizing complications. The approach is particularly useful in cases where conventional approaches have limitations [8,9]. Developed to facilitate extraperitoneal repair, eTEP allows hernia correction through small incisions, bypassing the peritoneal cavity to reduce the risk of intra-abdominal adhesions and postoperative pain. Studies by Belyansky et al. and Daes et al. describe the advantages of eTEP, noting reduced recovery times and fewer complications, especially when combined with enhanced recovery protocols [8]. Although both techniques are supported in specific cases, the EHS guidelines and current literature reflect limited direct comparisons of OS and eTEP under similar conditions, often focusing instead on isolated benefits within each technique.

To address this gap, the present study directly compares the OS and eTEP methods for ventral hernia repair, focusing on early clinical outcomes, complication rates, and patient recovery. To our knowledge, this is the first study to compare OS vs. eTEP for ventral hernia repair.

## 2. Materials and Methods

### 2.1. Study Design and Participants

Following approval from the Ethical Committee of the Carl von Ossietzky University Oldenburg (AZ 2024-095), a retrospective analysis was performed on a prospective dataset collected between July 2019 and December 2023. A comparative assessment of the short-term results of the two procedures was conducted after collecting the data. The study included all patients with ventral or incisional hernias who were treated with either the eTEP or the OS approach. In this study, the eTEP procedure was performed exclusively by a single surgeon with specialized laparoscopic expertise, whereas the OS technique was carried out by several different surgeons. All patients were scheduled for a follow-up appointment 30 days after surgery. For those who experienced complications prior to this date, an earlier follow-up was arranged.

### 2.2. Inclusion and Exclusion Criteria

All patients who underwent eTEP or OS repair for ventral abdominal wall defects were included in this study. The exclusion criteria were patients under 18 years of age, terminal illness, and other conditions making them unfit for surgery. The choice of procedure was made through a shared decision-making process between the surgeon and the patient, provided there were no absolute contraindications for either treatment. Absolute contraindications for eTEP included preoperative hernia defects larger than 7 cm in diameter and patients with previous mesh implantations, as well as patients with severe diastasis recti. Neither recurrent hernias nor loss of domain were exclusion criteria but might have been part of the patient/surgeon shared decision-making process for deciding in favor of either of the techniques. No exclusions were made based on gender, BMI, or chronic comorbidities, such as diabetes, COPD, or cardiac disease.

In our study, we focused exclusively on ventral hernias and therefore excluded cases classified as “L” (lateral) hernias according to the EHS classification. This decision was made to maintain a homogeneous study population, as lateral hernias differ significantly in anatomical location, surgical approach, and biomechanical characteristics compared to midline (M1–M5) hernias. Including both types could have introduced variability in outcomes and confounded comparisons between the eTEP and OS techniques, which are primarily optimized for midline defects. By limiting our analysis to ventral hernias, we aimed to ensure greater consistency and clinical relevance in the evaluation of these two surgical approaches [1,4].

**Figure 1:**
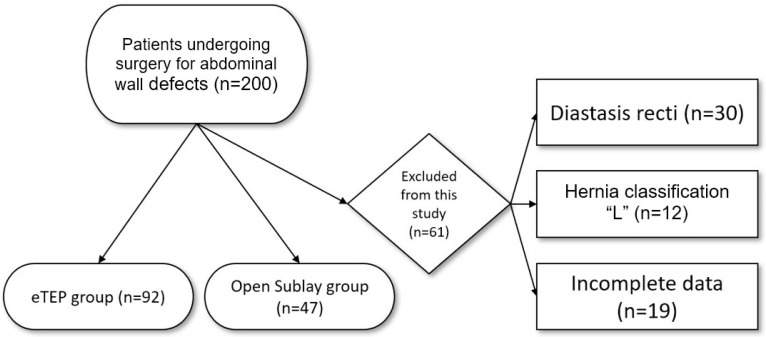
Flow diagram of patients undergoing eTEP vs. OS surgery.

Figure 1 represents the inclusion and exclusion criteria for patients undergoing eTEP and OS between 2019 and 2023. A total of 139 patients were included, with 92 patients undergoing eTEP and 47 patients undergoing OS (Figure 1).

Patients provided written consent for the use of their data in research. The study was approved by the university’s ethics commission.

### 2.3. Variables

The collected data included patient demographics (age, sex, and body mass index (BMI)) and comorbidities such as chronic obstructive pulmonary disease (COPD), cardiovascular diseases, diabetes, and smoking status. Perioperative physical status was defined using the American Society of Anesthesiologists (ASA) classification.

Hernias were categorized by locations according to the EHS classification, and their defect sizes were reported in square centimeters [3]. When hernias appeared at more than one anatomical site, each location was recorded individually, leading to a total number of hernias greater than the number of patients.

The reported surgical data included the date of surgery, its duration in minutes, intraoperative complications, and the size of the implanted mesh. Postoperative pain levels at rest were measured using the visual analog scale (VAS) on postoperative days 1–3. Additionally, the total admission period and postoperative complications, along with their respective Clavien–Dindo classifications, were reported.

Specific wound complications were categorized as surgical site infection (SSI), surgical site occurrence (SSO), and surgical site occurrence requiring procedural intervention (SSOPI). All infections, whether superficial or deep, were classified as SSI. SSOs included other non-infective surgical-site-specific complications, such as hematoma, seroma, wound drainage, delayed wound healing, wound dehiscence, necrosis, and early recurrences. Any SSI or SSO necessitating invasive intervention was further classified as SSOPI [10].

### 2.4. Surgical Techniques

#### 2.4.1. eTEP

The enhanced-view totally extraperitoneal (eTEP) technique utilizes small lateral incisions to access the bilateral retromuscular space while avoiding peritoneal entry, thereby reducing the risk of intra-abdominal adhesions. A continuous, tension-free anatomical space is created for placement of a synthetic mesh in the retrorectus plane, which is held in position by anatomical pressure rather than fixation with sutures or tacks. Surgical steps are adapted to individual anatomy and defect location, following minimally invasive principles for optimal tissue preservation and repair integrity.

A comprehensive, step-by-step description of the eTEP technique, accompanied by intraoperative images, is available in our previously published work [9].

#### 2.4.2. Open Sublay (OS)

The OS mesh technique is an open surgical procedure used for large incisional hernias. This method begins with a skin incision made directly over the protruding hernia sac during which the scar tissue is excised. The hernia sac is then opened, and any adhesions within the abdominal cavity are carefully released. The contents of the hernia sac are repositioned back into the abdominal cavity.

Next, the hernia sac is trimmed and removed along the edges of the hernia defect. The rectus sheath, which encloses the rectus abdominis muscle, is incised medially on both sides, and the submuscular spaces on the right and left side are dissected up to the linea semilunaris. The posterior layer is then sutured in the midline. A synthetic mesh is placed onto the posterior layer of the rectus sheath, behind the rectus abdominis muscle, and it is fixated at specific points to the posterior fascial layer using Prolene sutures [11]. Depending on the intraoperative findings, a Redon drain may be inserted. Finally, the anterior fascial layer is closed using a PDS suture. If the remaining defect between the two rectus abdominis muscles is still too large, a second mesh can be placed onto the anterior layer of the rectus sheath and fixed with sutures. This double mesh approach ensures a robust repair, providing additional support and reducing the likelihood of recurrence [12]. Throughout the procedure, meticulous hemostasis is maintained, and care is taken to minimize the risk of infection. Postoperative monitoring focuses on detecting any potential complications, such as seroma, hematoma, or infection, ensuring a smooth recovery and optimal surgical outcomes for the patient [13].

### 2.5. Statistical Analysis

Statistical analyses were conducted using SPSS (version 29). For continuous variables, we first generated histograms. Normally distributed data are presented as mean ± standard deviation (SD); non-normally distributed data are shown as median (interquartile range [IQR]). If the histograms were inconclusive, the Shapiro–Wilk test was used to confirm normality.

We divided the dataset into two groups: patients undergoing eTEP vs. patients undergoing OS. A Student’s *t*-test was used to compare normally distributed continuous variables; for non-normally distributed variables, the Mann–Whitney U-test was used. Categorical variables were compared via chi-square testing, with *p* < 0.05 being considered statistically significant.

In the event of missing data, variables were excluded pairwise. To reduce bias and ensure greater homogeneity within the patient population, a subgroup analysis focusing solely on primary hernias was conducted using identical statistical methods. Propensity score matching was attempted to further mitigate confounding factors, but the small sample size precluded acceptable matches (average caliper distances > 0.2) [14,15].

## 3. Results

### 3.1. Patient Demographics

A total of 139 patients were included: 92 in the eTEP group and 47 in the OS group. The mean age in the eTEP group was 51.82 years (range 18–84) vs. 54.96 years (range 20–81) in the OS group (*p* = 0.201). The mean BMI was 32.51 kg/m^2^ (range 21–57) for eTEP and 31.02 kg/m^2^ (range 19–65) for OS (*p* = 0.934). There was no significant difference in sex distribution; both groups showed a slight male predominance (63.04% vs. 63.83%).

The ASA scores trended higher in the OS group (*p* = 0.053). Smoking was more common in the OS group (31.91% vs. 20%; *p* = 0.144), but this was not statistically significant. Cardiovascular disease and obesity were the most common comorbidities in both groups (~50%). Median defect sizes were 6 cm^2^ in eTEP vs. 16 cm^2^ in OS (*p* = 0.028). Detailed demographics are shown in Table 1.

Umbilical hernias (M3) predominated in the eTEP group, whereas epigastric hernias (M2) were more frequent in the OS group. Table 2 lists the hernia types according to the EHS classification.

### 3.2. Intraoperative Findings

The key operative parameters included mesh size, defect size, operation time, postoperative pain, and hospital stay. Statistically significant differences were observed in mesh size (*p* < 0.001) and defect size (*p* = 0.028). Specifically, the eTEP group had larger mesh sizes, smaller defect sizes, lower pain scores on postoperative day 2, and shorter hospital stays.

The median operative time was 110 min (range 49–290) in the eTEP group vs. 100 min (range 45–230) in the OS group (*p* = 0.064), indicating no significant difference (Table 3). Intraoperative complications in the eTEP group included inadvertent peritoneal openings in two patients; in the OS group, three patients sustained serosal lesions.

**Figure 2:**
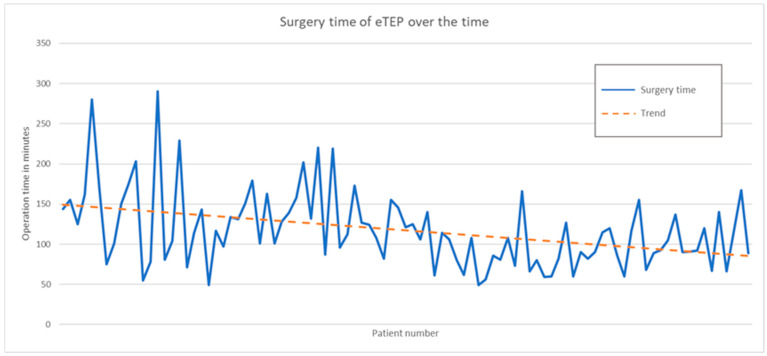
Decreasing time of eTEP surgery [9].

At the outset, considerable variation was noted in the duration of individual eTEP procedures, ranging from as short as 49 min to as long as 290 min. However, with an increasing number of cases, we observed a narrowing in the variability of surgery times among individual cases. Moreover, the trend indicated a general reduction in operative time, as depicted in Figure 2.

### 3.3. Postoperative Findings

The postoperative outcomes are summarized in Table 4. The overall complication rates were significantly lower in the eTEP group (6.52%) compared to OS (21.28%; *p* = 0.009). Using the Clavien–Dindo classification, minor complications (Grade I) were documented in two eTEP and five OS patients, while major complications (Grades IIIa/IIIb) occurred in three eTEP and four OS patients. One OS patient experienced a Grade V outcome unrelated to surgery; there were no deaths in the eTEP group.

The length of stay was significantly shorter in the eTEP group (median 3 days; IQR 1) than in the OS group (median 5 days; IQR 2; *p* < 0.001). Postoperative pain, measured using VAS, did not significantly differ between the groups: on day 1, the median VAS was 1.4 (IQR 1.92) in eTEP vs. 2 (IQR 2.75) in OS (*p* = 0.884); on day 2, 1 (IQR 2) vs. 1.3 (IQR 1.9) (*p* = 0.208); and on day 3, 0.5 (IQR 2) vs. 0 (IQR 2) (*p* = 0.636).

Table 5 details the types of postoperative complications. In the eTEP group, notable complications included hemoglobin-relevant bleeding (n = 2), abscess formation (n = 1), early recurrence (n = 1), and hematoma (n = 2). In the OS group, complications included wound dehiscence (n = 1), ruptured drainage tube (n = 1), wound infections (n = 4), hematoma (n = 2), pneumonia (n = 1), and subcutaneous seroma (n = 1).

## 4. Discussion

Managing midline or ventral hernias has historically posed challenges, prompting the development of numerous surgical techniques. Recently, minimally invasive procedures have gained traction due to advantages such as reduced wound infection risks and shorter hospital stays, as highlighted by the European Hernia Society’s guidelines [4]. Despite the variety of these procedures, the optimal method regarding safety and feasibility remains uncertain. The guidelines recognize the potential benefits of laparoscopic methods and retrorectus mesh placement but call for more comprehensive data to make definitive recommendations [3]. Therefore, continued research comparing different treatments is essential for developing evidence-based guidelines for ventral hernia management.

The eTEP procedure represents a relatively recent and promising technique for ventral hernia repair, developed as an evolution of the Rives–Stoppa method introduced in the 1960s [16]. Originally designed for open inguinal hernia repair, it has been adapted for minimally invasive surgery and other hernia types. There is limited literature on eTEP for ventral hernias, particularly in Germany. One study by Bauer et al. analyzed 61 robotic-assisted eTEP procedures between 2019 and 2022 [17].

Despite the growing interest in minimally invasive techniques like the eTEP approach and the long-established use of the OS method in ventral hernia repair, there are no studies directly comparing these two techniques. This lack of comparative research highlights a significant gap in the current literature, as both methods are widely utilized and supported by clinical guidelines for specific indications.

This study seeks to address this gap by providing a direct comparison of eTEP and OS approaches in ventral hernia repair, focusing on early clinical outcomes, complication rates, and patient recovery. As the first study to directly evaluate these techniques side by side, it represents a preliminary step toward a more comprehensive understanding of their relative benefits and limitations.

Several European studies have compared the OS technique with more modern surgical approaches for ventral hernia repair. The findings suggest that the OS procedure may be associated with increased postoperative pain, longer hospital stays, and a higher incidence of complications. For instance, a large-scale analysis utilizing data from the Herniamed Hernia Registry compared laparoscopic intraperitoneal onlay mesh (IPOM) repair with the OS technique in elective incisional hernia surgeries. The study found that the OS approach resulted in significantly higher rates of postoperative complications (10.5% vs. 3.4%; *p* < 0.001), including surgical site infections, seromas, and bleeding. Additionally, patients undergoing the OS procedure experienced longer hospital stays compared to those who had laparoscopic IPOM repair (6.14 ± 5.29 days vs. 4.35 ± 3.32 days; *p* < 0.001) [18].

Conducted as a retrospective analysis, our study provides valuable insights into real-world outcomes between eTEP and OS surgery, a comparison that is under-represented in the existing literature. Most existing comparable studies mentioned above also have small sample sizes, a limitation we mitigated through the use of our larger dataset. Additionally, the comparison between laparoscopic IPOM and OS repair is inherently challenging due to differences in mesh placement within distinct anatomical layers, which may introduce potential bias.

Our findings indicate that the eTEP approach demonstrates both safety and feasibility in the management of ventral hernias comparable to OS and potentially superior in some aspects. Consistent with previous research, our study showed shorter hospital stays for eTEP patients compared to OS patients, with a median stay of 3 days for eTEP and 5 days for OS. Shorter hospital stays lead to cost savings and quicker return to normal activities, reducing the risk of complications associated with prolonged hospitalization [19,20]. The marginal non-significant increased operative time was outweighed by the benefits mentioned. Patients were released significantly earlier, and although not statistically significant, the postoperative pain was lower on days 1 and 2 in the eTEP group.

While our study did not find a statistically significant difference in early postoperative pain between eTEP and OS, the average pain scores were lower in the eTEP group on the first and second postoperative days. This aligns with the literature suggesting less postoperative pain in eTEP due to the mesh being held in place by the rectus sheath rather than anchors [21,22]. The rate of post-surgical adverse events was lower in the eTEP group, becoming statistically significant in the subgroup analysis.

Our results also showed a slightly longer average operation time for eTEP, whereas OS showed a shorter average surgery time. This aligns with most previous studies and suggests that eTEP procedures generally take longer, possibly due to the novelty of the technique and the learning curve for surgeons [23,24].

In the context of minimally invasive ventral hernia repair, a study by Wieland et al. provides important insights. The retrospective cohort study compared eTEP with IPOM in ventral hernia repair [9]. The study concluded that eTEP is a safe and effective alternative to IPOM, with advantages such as shorter hospital stays and a potentially lower complication rate, despite requiring longer operative times. Furthermore, eTEP patients reported lower postoperative pain levels, although this difference did not reach statistical significance.

In summary, our study supports the safety and feasibility of eTEP for ventral hernias, with potential advantages over OS in terms of hospital stay and postoperative complications. However, further research, particularly randomized controlled trials, is needed to comprehensively evaluate these findings and establish more definitive recommendations, potentially leading to guideline updates. The evaluation of long-term outcomes can add valuable data for implementation in the clinical daily routine and will take place in future studies from our clinic. This study concentrates solely on early outcomes to prove safety and feasibility.

### Limitations and Strengths

The study has several limitations that must be acknowledged. First, it relies on retrospective data, which may introduce selection bias and limit the ability to control for confounding variables. Second, the sample size is relatively small, which could reduce the statistical power of the findings and the generalizability of the results. Additionally, in the eTEP group, all procedures were performed by a single surgeon, while the sublay group included surgeries conducted by multiple surgeons, potentially introducing variability in surgical technique and outcomes. However, the study also has notable strengths. The use of a single surgeon for the eTEP group eliminates inter-surgeon variability, thereby reducing bias and allowing for a more consistent assessment of this specific technique. Moreover, this study is among the first to directly compare the eTEP and OS approaches, contributing valuable insights to the literature and providing a foundation for further research in this area. Future directions should include larger sample sizes, if necessary, in multicenter studies. Also, randomized controlled studies are needed to yield more robust results.

The use of propensity score matching was initiated to minimize potential confounding factors and ensure a more robust comparison between groups.

## 5. Conclusions

Our findings show that eTEP is a safe and feasible surgical procedure for ventral hernias. Compared to the OS technique, eTEP offers the benefit of a shorter postoperative hospital stay. Additionally, our findings suggest that eTEP is associated with a lower postoperative complication rate. Although eTEP patients may experience less postoperative pain, our study did not provide statistically significant evidence to confirm this. Larger datasets are crucial for comparisons of this nature, as they enhance the robustness and reliability of the analysis, improving the interpretability of the results.

## Figures and Tables

**Table 1:** Full study group—Demographics.

Variables	eTEP (n = 92)	OS (n = 47)	*p*-Value
Age (years)	51.82 ± (13.15) *	54.96 ± (15.11) *	0.28
BMI (kg/cm^2^)	32.51 ± (7.45) *	31.02 ± (7.92) *	0.41
Obesity level			
Normal weight	10	8	0.45
Overweight	25	13	1.0
Obesity level I	26	12	0.888
Obesity level II	17	10	0.867
Obesity level III	14	4	0.397
Sex			0.927
Male	58; 63.04%	30; 63.83%	
Female	34; 36.96%	17; 36.17%	
ASA Score			
I	6; 6.52%	5; 10.64%	0.604
II	62; 67.39%	25; 53.19%	0.147
III	24; 26.09%	14; 29.79%	0.793
IV	0	3; 6.38	0.067
V	0	0	/
Risk factors			
Cardiovascular disease	48; 52.17%	21; 44.68%	0.403
COPD (%)	2; 2.17%	1; 2.13%	0.986
Diabetes (%)	11; 11.96%	6; 12.77%	0.890
Smoking (%)	19; 20.0%	15; 31.91%	0.144
Defect size (cm^2^)	6 (IQR 9.25) ^†^	16 (IQR 40) ^†^	**0.028**

* = Mean + SD; ^†^ = Median + IQR; BMI = Body mass index; ASA = American Society of Anesthesiologists; COPD = Chronic obstructive pulmonary disease.

**Table 2:** Hernia type subgroup analysis.

Classification	eTEP (n = 127)	OS (n = 50)	*p*-Value
M1	2, 2.11%	2, 4.26%	0.548
M2	41, 44.57%	21, 44.68%	0.990
M3	73, 79.35%	18, 38.3%	**<0.001**
M4	8, 8.70%	7, 14.89%	0.265
M5	3, 3.26%	2, 4.26%	0.842

Please note that a single patient can present with multiple hernia types, thus resulting in total percentages exceeding 100%.

**Table 3:** Intraoperative findings.

Variables	eTEP (n = 92)	OS (n = 47)	*p*-Value
Mesh size (cm^2^)	450 (IQR 125) ^†^	150 (IQR 270) ^†^	**<0.001**
Operation time (minutes)	110 (IQR 60) ^†^	100 (IQR 47) ^†^	0.064
Intraoperative complications	2 (2.17%)	3 (6.38%)	0.207
Admission period (days)	3 (IQR 1) ^†^	5 (IQR 2) ^†^	**<0.001**

^†^ = Median + IQR.

**Table 4:** Postoperative outcomes and complications.

Variables	eTEP (n = 92)	OS (n = 47)	*p*-Value
Postoperative complications	6 (6.52%)	10 (21.28%)	**0.009**
Clavien–Dindo			0.082
I	2	5	
II	1	0	
IIIa	1	0	
IIIb	2	4	
IV	0	0	
V	0	1	
Admission period (days)	3 (IQR 1) ^†^	5 (IQR 2) ^†^	**<0.001**
VAS			
Postoperative day 1	1.4 (IQR 1,92) ^†^	2 (IQR 2.75) ^†^	0.884
Postoperative day 2	1 (IQR 2) ^†^	1.3 (IQR 1.9) ^†^	0.208
Postoperative day 3	0.5 (IQR 2) ^†^	0 (IQR 2) ^†^	0.636

^†^ = Median + IQR; VAS = Visual Analog Scale.

**Table 5:** Postoperative complications in detail.

Technique	n	Description of Complication
eTEP	2	Hemoglobin-relevant bleeding
	1	Abscess
	1	Early recurrence
	2	Hematoma
OS	1	Wound dehiscence
	1	Rupture of a drainage tube
	4	Infection of the wound
	2	Hematoma
	1	Pneumonia
	1	Subcutaneous seroma

## Data Availability

Due to national data regulations, the data are only available upon request.

## Abbreviations

The following abbreviations are used in this manuscript:

eTEP	Enhanced-View Totally Extraperitoneal
OS	Open Sublay
VAS	Visual Analog Scale
BMI	Body Mass Index
COPD	Chronic Obstructive Pulmonary Disease
ASA	American Society of Anesthesiologists
SSI	Surgical Site Infection
SSO	Surgical Site Occurrence
SSOPI	Surgical Site Occurrence Requiring Procedural Intervention
